# Potential influence of omentin-1 genetic variants on the clinicopathological features of patients with hepatocellular carcinoma

**DOI:** 10.7150/jca.128357

**Published:** 2026-02-18

**Authors:** Sung-Lin Hu, Hsiang-Lin Lee, Ming-Yu Lien, Edie-Rosmin Wu, Shun-Fa Yang, Chih-Hsin Tang

**Affiliations:** 1 School of Medicine, China Medical University, Taichung, Taiwan.; 2 Department of Family Medicine, China Medical University Hsinchu Hospital, Hsinchu, Taiwan.; 3 School of Medicine, Chung Shan Medical University, Taichung, Taiwan.; 4 Department of Surgery, Chung Shan Medical University Hospital, Taichung, Taiwan.; 5 Division of Hematology and Oncology, Department of Internal Medicine, China Medical University Hospital, Taichung, Taiwan.; 6 Division of General Surgery, Changhua Christian Hospital, Changhua, Taiwan.; 7 Institute of Medicine, Chung Shan Medical University, Taichung, Taiwan.; 8 Department of Medical Research, Chung Shan Medical University Hospital, Taichung, Taiwan.; 9 Department of Pharmacology, School of Medicine, China Medical University, Taichung, Taiwan.; 10 Department of Medical Laboratory Science and Biotechnology, Asia University, Taichung, Taiwan.; 11 Chinese Medicine Research Center, China Medical University, Taichung, Taiwan.

**Keywords:** Omentin-1, Hepatocellular carcinoma, genetic polymorphisms, rs79209815

## Abstract

Hepatocellular carcinoma (HCC) ranks as the fifth supreme prevalent cancer within men globally and the ninth among female, serving as a significant contributor to cancer-associated deaths. The adipokine omentin-1 has been demonstrated to have a defensive effect by decreasing the secretion of proinflammatory cytokines. The connections among lifestyle factors that promote cancer, *OMNT1* polymorphisms, and HCC are still not well understood. Our investigation focused on the influence of clinicopathological characteristics and four variants of the *OMNT1* gene (rs2274907, rs35779394, rs4656959, and rs79209815) on healthy controls as well as Taiwanese individuals with HCC. According to our data, individuals with the *OMNT1* rs79209815 variant (TC or CC genotypes) are at an elevated risk of progressing to stage III/IV disease and larger tumors than those with the TT genotype. Males exhibited these associations more prominently than females. Moreover, *OMNT1* expression levels were markedly reduced in individuals with the wild-type TT homozygous genotype when compared to those with the TC or CC genotypes of rs79209815. The complexity of genetic influences on HCC is highlighted by our study, which suggests that *OMNT1* polymorphisms may have an impact on tumor stage and progression.

## Introduction

Hepatocellular carcinoma (HCC) ranks as the fifth supreme prevalent cancer within men globally and the ninth among female, serving as a significant contributor to cancer-related deaths 1. HCC correlates with a low five-year survival rate and rising mortality rates 2, 3. In Taiwan, the second most common reason of cancer-related fatalities is HCC 4, 5. Genetic variation is crucial for susceptibility to HCC and its progression. Most individuals who encounter the recognized infectious or environmental risk mediators (such as alcohol misuse, hepatitis virus infection, or non-alcoholic fatty liver disorder linked to insulin resistance, diabetes or obesity) do not go on to progress HCC. This implies that personal susceptibility plays a role in tumorigenesis 6, 7. Data on the frequency of genotype distribution can help to chart single nucleotide polymorphism (SNP) diversity within a population and investigate the risk and progression of certain disorders 8, 9. Investigations that have recently come to light suggest a link between SNPs in specific genes and the vulnerability to and clinicopathological status of HCC.

Adipokines are bioactive agents secreted by adipose tissue that regulate a range of physiological processes, including inflammation, homeostasis, insulin responsiveness, and immune responses 10, 11. Metabolic and inflammatory pathways are influenced by key adipokines for instance omentin-1, adiponectin, resistin and leptin, which act locally or systemically through endocrine, autocrine, or paracrine processes 12. Adipokines have multifaceted effects in cancer, impacting the tumor microenvironment, cellular proliferation, apoptosis, angiogenesis, and metastasis 13. Omentin-1 have identified adipokine consisting of 313 amino acids, is primarily generated in the small intestine as well as in adipose tissue and omental 14, 15. Omentin-1 exhibits anti-hyperinsulinemic and anti-inflammatory functions 16. It has been documented to play a protective effect in decreasing proinflammatory cytokine generation 17. Previous experimental studies have shown that omentin-1 is positively linked with increased levels of anti-inflammatory mediators 18, 19. In the context of cancer, there is an inverse relationship between serum omentin-1 levels and obesity, indicating that omentin-1 could be a marker for cancer development 20. Moreover, omentin-1 might act as a tumor-suppressor mediator, given that patients with renal cell carcinoma have been observed to have lower serum levels of omentin-1 21. No data are available on the links between carcinogenic lifestyle factors, *OMNT1* gene polymorphisms, and HCC. Consequently, this research investigated the impact of carcinogenic lifestyle factors and *OMNT1* gene polymorphisms on the likelihood of HCC development in a cohort of Taiwanese. We also investigated the relationships between *OMNT1* genotypes and the histopathological prognostic variables of HCC.

## Materials and Methods

### Study participants

We registered 413 patients with HCC at Chung Shan Medical University Hospital in Taiwan. From the Taiwan Biobank Project, 826 healthy controls (HCs) with no cancer history were randomly chosen and anonymized. Every participant in the study belonged to the Han Chinese ethnicity. Patients with HCC were staged using the 2010 American Joint Committee on Cancer (AJCC) TNM staging system, which takes into account tumor morphology, the number of affected lymph nodes, and metastases 22. Prior to entering the study, each participant gave informed written consent and filled out a structured questionnaire regarding their sociodemographic status, as well as their use of alcohol and cigarettes. A diagnosis of liver cirrhosis was made based on biopsy results, suitable sagittal CT or MRI scans, or biochemical indicators of liver parenchymal damage accompanied by endoscopic esophageal or gastric varices. Before the study began, it received approval from the Institutional Review Board of Chung Shan Medical University Hospital.

### Selection and genotyping of SNPs

The *OMNT1* SNPs rs2274907, rs35779394, rs4656959, and rs79209815 were selected based on previous reports 23, 24. Every SNP had a minor allele frequency greater than 5%. Genomic DNA was extracted from 3 mL peripheral blood samples using QIAamp DNA Blood Kits (Qiagen, CA, USA). Using previously described assessment techniques 8, 20, 21, allelic discrimination was performed on the SNPs. RT-qPCR experiments and the isolation of RNA were performed following the protocols we published earlier 25, 26.

### Analysis of clinical dataset

The GTEx portal (gtexportal.org/home/) serves as a comprehensive public resource for the analysis of gene levels and modulation specific to various tissues. It provides quantitative trait loci (QTLs), histological images, and gene expression data that is open-access 27.

### Statistical analysis

To assess the differences between the HCC and control groups, the Fisher's exact test and Mann-Whitney U test were employed, with *p*-values below 0.05 considered statistically significant. Logistic regression was used to calculate odds ratios (ORs) and their 95% confidence intervals (CIs) for the associations between genotype frequencies and HCC risk. The collected data were analyzed using version 9.1 of the Statistical Analytic System (SAS) software.

## Results

The demographic characteristics of the 413 patients with HCC and the 826 cancer-free HCs) did not show significant differences (Table 1). Controls reported alcohol consumption significantly less often than patients (*p* < 0.001), but there was no difference in cigarette smoking status between the two groups (*p* = 0.409) (Table 1). The proportions of HCC patients who tested positive for HBsAg (44.8%) and anti- HCV antibodies (36.6%) (Table 1). At the time of enrollment in the study, 310 patients (75.1%) presented with stage I/II HCC, while 103 (24.9%) had stage III/IV disease. Patients with HCC also presented with N1+N2+N3 lymph node status (2.2%), metastasis (5.8%) and vascular invasion (12.3%). Liver cirrhosis was present in most patients (86.2%) (Table 1).

Genotyping results for the *OMNT1* SNPs in HCs and HCC patients are shown in Table 2. The homozygous T/T alleles for rs2274907, rs35779394, and rs79209815, as well as the homozygous A/A allele for rs4656959, were the most common (Table 2). After adjusting for age, gender, cigarette smoking, and alcohol consumption (Table 2), none of the genotypes for the four *OMNT1* SNPs across different groups exhibited notable associations.

Next, we conducted a comparison of the distributions of clinical aspects and *OMNT1* genotypes among HCC patients. No significant influence of SNPs rs2274907, rs35779394 and rs4656959 on clinicopathologic traits in HCC patients (Table 3). However, compared with patients with the T/T genotype, those with at least one polymorphic C allele at the rs79209815 SNP (T/C+C/C genotype) were susceptible to progressing to stage III/IV disease (OR, 1.899; 95% CI, 1.027~3.512; *p*<0.05) and large tumors (OR, 2.055; 95% CI, 1.109~3.810; *p*<0.05) (Table 4). The TC or CC genotypes at rs79209815 were related with an increased risk of stage III/IV disease (OR, 2.298; 95% CI, 1.142-4.622; *p* < 0.05) and larger tumors (OR, 2.298; 95% CI, 1.142-4.622; *p* < 0.05) in male, but not female, patients compared with the TT genotype (Table 5).

**Figure 1:**
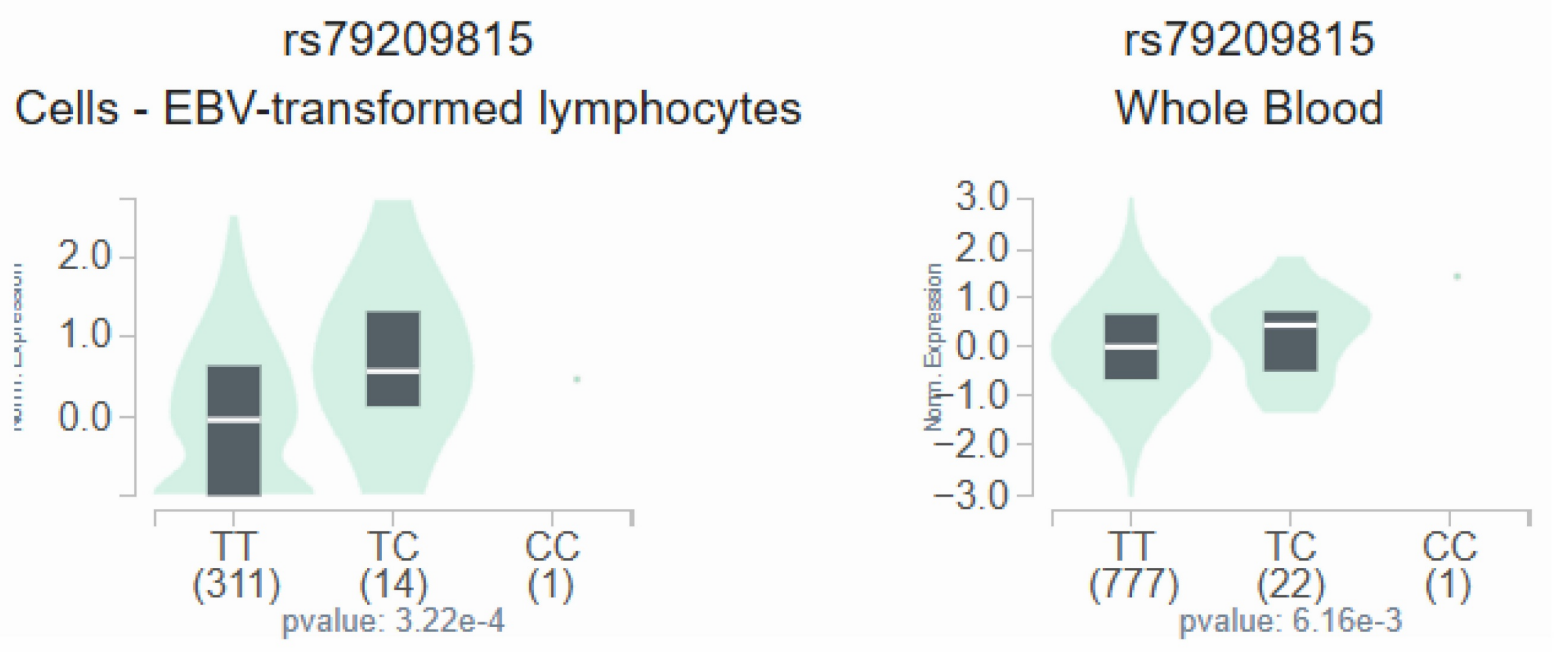
The *OMNT1* shows a substantial eQTL association with rs79209815 genotypes in lymphocytes and whole blood samples from the GTEx database.

GTEx data indicate that, in lymphocytes and whole blood, individuals carrying the C variant at rs79209815 showed a trend toward augmented omentin-1 expression compared with those with the wild-type TT homozygous genotype (Figure 1).

## Discussion

Many cancer reports have confirmed the effectiveness of biomarkers based on genetic aberrations related to tumors in assessing risk, aiding in early diagnosis, and predicting treatment results 28, 29. Approximately 1% of the overall population carries genetic polymorphisms, which are variations in genomic sequences among individuals. Repetitive sequences most frequently exhibit alterations in the form of SNPs 30. An expanding body of investigation has recently underscored the significance of SNPs and other genetic changes in predicting and defining pharmacotherapeutic functions in HCC 31, 32. Moreover, the methodical identification of functional variants linked to cancer risk has demonstrated how SNPs in functional domains affect gene level and tumor susceptibility, highlighting the importance of SNPs in tumor biology 33. These findings are supplemented by thorough reviews that detail the biological and molecular processes through which SNPs affect gene expression, thereby affecting the progression and development of tumor 34. Hypothesis-driven genetic research has informed both case-control and prospective cohort reports that investigated the connection between SNPs and HCC. The studies have underscored the connection between changes impacting multiple biological mechanisms—for instance oxidative stress, DNA repair, and inflammation processes—and the evolution of liver cancer in patients with hepatitis 35. We investigated polymorphisms in the *OMNT1* gene and noted their different distributions among HCC patients. Our investigation showed that patients with the T/C+C/C genotypes of rs79209815 exhibited a significantly elevated risk of progressing stage III/IV disease and large tumors.

Adipokines are unique bioactive peptides released by adipose tissues and play a role in various bodily functions 36, 37. To examine the function of adipose tissue in the progression of inflammation and carcinogenesis, many investigators have been investigating this issue for the last two decades 38, 39. Recent studies have documented that omentin-1 plays a vital effect in cell differentiation and the promotion of cancer cell death 40. Many associated studies found that the levels of omentin-1 in circulation among patients with colorectal and renal cell carcinoma differed, suggesting that omentin-1 might play a part in cancer development 41. Recently, omentin-1 SNPs have been investigated in several cancers. For example, in OSCC, the TA and AA genotypes of SNP rs2274907 were related with an augmented risk of progressing to an advanced clinical stage compared with the TT genotype 23. The *OMNT1* rs2274907 and rs4656959 variants are protective against perineural invasion, particularly in prostate cancer patients without biochemical recurrence 24. Conversely, research on omentin-1 and HCC is limited. We conducted this study to compare the allelic distributions of *OMNT1* gene polymorphisms between HCs and HCC patients. Carriers of at least one C allele (genotypes TC or CC) at the *OMNT1* SNP rs79209815 exhibited a heightened risk for developing stage III/IV disease and larger tumors, according to our findings. It is worth mentioning that these correlations were more marked in male patients than in female ones. Furthermore, there was a tendency for the omentin-1 expression to be higher in individuals with the C variant at rs79209815 than in those with the wild-type TT homozygous genotype. Omentin-1 levels are thus crucially linked to the advancement of HCC in patients. It is important to note the limitations of the current study. More research is required, which calls for a larger sample size and a longer follow-up time. Furthermore, an independent cohort of HCC cases from Taiwanese communities and other cohorts found in open-access databases must be used to confirm the current findings.

To sum up, our study is the first to uncover links between *OMNT1* gene variants and HCC. The *OMNT1* rs79209815 variant (genotypes TC or CC) is linked to a heightened risk of developing stage III/IV disease and larger tumors, especially among male HCC patients, as our findings suggest. Moreover, the wild-type TT homozygous genotype was linked to considerably reduced *OMNT1* expression levels in comparison to the TC or CC genotypes of rs79209815, suggesting that this *OMNT1* SNP plays a crucial role in HCC development.

## Figures and Tables

**Table 1:** The distributions of demographical characteristics in 826 controls and 413 patients with HCC.

Variable	Controls (N=826)	Patients (N=413)	p value
Age (yrs)			
<60	322 (39.0%)	140 (33.9%)	p = 0.081
≥ 60	504 (61.0%)	273 (66.1%)	
Gender			
Male	612 (74.1%)	289 (70.0%)	p = 0.125
Female	214 (25.9%)	124 (30.0%)	
Cigarette smoking			
No	502 (60.8%)	261 (63.2%)	p = 0.409
Yes	324 (39.2%)	152 (36.8%)	
Alcohol drinking			
No	701 (84.9%)	284 (68.8%)	p < 0.001*
Yes	125 (15.1%)	129 (31.2%)	
HBsAg			
Negative		228 (55.2%)	
Positive		185 (44.8%)	
Anti-HCV			
Negative		262 (63.4%)	
Positive		151 (36.6%)	
Stage			
I+II		310 (75.1%)	
III+IV		103 (24.9%)	
Tumor T status			
T1+T2		315 (76.3%)	
T3+T4		98 (23.7%)	
Lymph node status			
N0		404 (97.8%)	
N1+N2+N3		9 (2.2%)	
Metastasis			
M0		389 (94.2%)	
M1		24 (5.8%)	
Vascular invasion			
No		362 (87.7%)	
Yes		51 (12.3%)	
Liver cirrhosis			
Negative		57 (13.8%)	
Positive		356 (86.2%)	

* *p* value < 0.05 as statistically significant.

**Table 2:** Genotyping and allele frequency of *OMNT1* single nucleotide polymorphism (SNP) in HCC and normal controls.

Variable	Controls (N=826) (%)	Patients (N=413) (%)	AOR (95% CI)	p value
**rs2274907**				
TT	367 (44.4%)	181 (43.8%)	1.000 (reference)	
TA	377 (45.6%)	189 (45.8%)	0.998 (0.772~1.291)	p=0.989
AA	82 (10.0%)	43 (10.4%)	1.091 (0.715~1.664)	p=0.687
TA+AA	459 (55.6%)	232 (56.2%)	1.014 (0.794~1.296)	p=0.909
**rs35779394**				
TT	645 (78.1%)	310 (75.1%)	1.000 (reference)	
TC	166 (20.1%)	92 (22.3%)	1.186 (0.879~1.599)	p=0.265
CC	15 (1.8%)	11 (2.7%)	1.547 (0.689~3.473)	p=0.291
TC+CC	181 (21.9%)	103 (24.9%)	1.216 (0.913~1.622)	p=0.182
**rs4656959**				
AA	379 (45.9%)	186 (45.0%)	1.000 (reference)	
AG	367 (44.4%)	185 (44.8%)	1.010 (0.781~1.305)	p=0.939
GG	80 (9.7%)	42 (10.2%)	1.083 (0.708~1.658)	p=0.713
AG+GG	447 (54.1%)	227 (55.0%)	1.023 (0.801~1.306)	p=0.856
**rs79209815**				
TT	708 (85.7%)	361 (87.4%)	1.000 (reference)	
TC	112 (13.6%)	45 (10.9%)	0.783 (0.535~1.145)	p=0.207
CC	6 (0.7%)	7 (1.7%)	2.327 (0.756~7.157)	p=0.141
TC+CC	118 (14.3%)	52 (12.6%)	0.861 (0.600~1.236)	p=0.419

Adjusted for the effects of age, gender, cigarette smoking and alcohol drinking.

**Table 3:** Odds ratios (ORs) and 95% confidence intervals (CIs) of the clinical status and *OMNT1* rs2274907 and rs35779394 genotypic frequencies in 413 patients with HCC.

Variable	rs2274907				rs35779394			
	TT (*N*=181)	TA+AA (*N*=232)	OR (95% CI)	p value	TT (N=310)	TC+CC (N=103)	OR (95% CI)	p value
**Clinical Stage**								
Stage I/II	139 (76.8%)	171 (73.7%)	1.000	0.472	233 (75.2%)	77 (74.8%)	1.000	0.935
Stage III/IV	42 (23.2%)	61 (26.3%)	1.181 (0.751~1.856)		77 (24.8%)	26 (25.2%)	1.022 (0.611~1.708)	
**Tumor size**								
T1+T2	141 (77.9%)	174 (75.0%)	1.000	0.492	238 (76.8%)	77 (74.8%)	1.000	0.677
T3+T4	40 (22.1%)	58 (25.0%)	1.175 (0.742~1.861)		72 (23.2%)	26 (25.2%)	1.116 (0.666~1.872)	
**Lymph node metastasis**								
No	179 (98.9%)	225 (97.0%)	1.000	0.187	303 (97.7%)	101 (98.1%)	1.000	0.849
Yes	2 (1.1%)	7 (3.0%)	2.784 (0.571~13.568)		7 (2.3%)	2 (1.9%)	0.857 (0.175~4.193)	
**Distant metastasis**								
No	173 (95.6%)	216 (93.1%)	1.000	0.286	289 (93.2%)	100 (97.1%)	1.000	0.147
Yes	8 (4.4%)	16 (6.9%)	1.602 (0.670~3.831)		21 (6.8%)	3 (2.9%)	0.413 (0.121~1.414)	
**Vascular invasion**								
No	157 (86.7%)	205 (88.4%)	1.000	0.619	269 (86.8%)	93 (90.3%)	1.000	0.347
Yes	24 (13.3%)	27 (11.6%)	0.862 (0.479~1.551)		41 (13.2%)	10 (9.7%)	0.705 (0.340~1.464)	
**HBsAg**								
Negative	94 (51.9%)	134 (57.8%)	1.000	0.238	171 (55.2%)	57 (55.3%)	1.000	0.975
Positive	87 (48.1%)	98 (42.2%)	0.790 (0.534~1.168)		139 (44.8%)	46 (44.7%)	0.993 (0.634~1.554)	
**Anti-HCV**								
Negative	116 (64.1%)	146 (62.9%)	1.000	0.809	198 (63.9%)	64 (62.1%)	1.000	0.751
Positive	65 (35.9%)	86 (37.1%)	1.051 (0.702~1.574)		112 (36.1%)	39 (37.9%)	1.077 (0.680~1.708)	
**Liver cirrhosis**								
Negative	25 (13.8%)	32 (13.8%)	1.000	0.996	45 (14.5%)	12 (11.7%)	1.000	0.465
Positive	156 (86.2%)	200 (86.2%)	1.002 (0.570~1.760)		265 (85.5%)	91 (88.3%)	1.288 (0.652~2.541)	

ORs with their 95% CIs were estimated by logistic regression models.

**Table 4:** Odds ratios (ORs) and 95% confidence intervals (CIs) of the clinical status and *OMNT1* rs4656959 and rs79209815 genotypic frequencies in 413 patients with HCC.

Variable	rs4656959				rs79209815			
	AA (N=186)	AG+GG (N=227)	OR (95% CI)	p value	TT (N=361)	TC+CC (N=52)	OR (95% CI)	p value
**Clinical Stage**								
Stage I/II	141 (75.8%)	169 (74.4%)	1.000	0.751	277 (76.7%)	33 (74.4%)	1.000	0.039*
Stage III/IV	45 (24.2%)	58 (25.6%)	1.075 (0.686~1.685)		84 (23.3%)	19 (25.6%)	**1.899 (1.027~3.512)**	
**Tumor size**								
T1+T2	143 (76.9%)	172 (75.8%)	1.000	0.792	282 (78.1%)	33 (63.5%)	1.000	0.020*
T3+T4	43 (23.1%)	55 (24.2%)	1.063 (0.674~1.679)		79 (21.9%)	19 (36.5%)	**2.055 (1.109~3.810)**	
**Lymph node metastasis**								
No	183 (98.4%)	221 (97.7%)	1.000	0.476	354 (98.1%)	50 (96.2%)	1.000	0.379
Yes	3 (1.6%)	6 (2.6%)	1.656 (0.409~6.714)		7 (1.9%)	2 (3.8%)	2.023 (0.409~10.010)	
**Distant metastasis**								
No	177 (95.2%)	212 (93.4%)	1.000	0.444	338 (93.6%)	51 (98.1%)	1.000	0.200
Yes	9 (4.8%)	15 (6.6%)	1.392 (0.595~3.256)		23 (6.4%)	1 (1.9%)	0.288 (0.038~2.180)	
**Vascular invasion**								
No	161 (86.6%)	201 (88.5%)	1.000	0.541	318 (88.1%)	44 (84.6%)	1.000	0.477
Yes	25 (13.4%)	26 (11.5%)	0.833 (0.463~1.498)		43 (11.9%)	8 (15.4%)	1.345 (0.593~3.046)	
**HBsAg**								
Negative	98 (52.7%)	130 (57.3%)	1.000	0.352	201 (55.7%)	27 (51.9%)	1.000	0.611
Positive	88 (47.3%)	97 (42.7%)	0.831 (0.563~1.227)		160 (44.3%)	25 (48.1%)	1.163 (0.650~2.082)	
**Anti-HCV**								
Negative	118 (63.4%)	144 (63.4%)	1.000	0.999	224 (62.0%)	38 (73.1%)	1.000	0.123
Positive	68 (36.6%)	83 (36.6%)	1.000 (0.669~1.496)		137 (38.0%)	14 (26.9%)	0.602 (0.315~1.152)	
**Liver cirrhosis**								
Negative	26 (14.0%)	31 (13.7%)	1.000	0.925	48 (13.3%)	9 (17.3%)	1.000	0.433
Positive	160 (86.0%)	196 (86.3%)	1.027 (0.586~1.801)		313 (86.7%)	43 (82.7%)	0.733 (0.336~1.598)	

ORs with their 95% CIs were estimated by logistic regression models. * p < 0.05 as statistically significant.

**Table 5:** The odds ratio (OR) and 95% confidence interval (CI) for clinical status and the genotypic frequencies of *OMNT1* rs79209815 in hepatocellular carcinoma (HCC) patients stratified by gender.

Variable	Male (N=289)				Female (N=124)			
	TT (N=249)	TC+CC (N=40)	OR (95% CI)	p value	TT (N=112)	TC+CC (N=12)	OR (95% CI)	p value
**Clinical Stage**								
Stage I/II	193 (77.5%)	24 (60.0%)	1.000	0.017*	84 (75.0%)	9 (75.0%)	1.000	1.000
Stage III/IV	56 (22.5%)	16 (40.0%)	**2.298 (1.142~4.622)**		28 (25.0%)	3 (25.0%)	1.000 (0.253~3.955)	
**Tumor size**								
T1+T2	193 (77.5%)	24 (60.0%)	1.000	0.017*	89 (79.5%)	9 (75.0%)	1.000	0.718
T3+T4	56 (22.5%)	16 (40.0%)	**2.298 (1.142~4.622)**		23 (20.5%)	3 (25.0%)	1.290 (0.323~5.151)	
**Lymph node metastasis**								
No	244 (98.0%)	38 (95.0%)	1.000	0.253	110 (98.2%)	12 (100.0%)	1.000	0.641
Yes	5 (2.0%)	2 (5.0%)	2.568 (0.481~13.713)		2 (1.8%)	0 (0.0%)	----	
**Distant metastasis**								
No	232 (93.2%)	39 (97.5%)	1.000	0.293	106 (94.6%)	12 (100.0%)	1.000	0.411
Yes	17 (6.8%)	1 (2.5%)	0.350 (0.045~2.705)		6 (5.4%)	0 (0.0%)	----	
**Vascular invasion**								
No	217 (87.1%)	33 (82.5%)	1.000	0.424	101 (90.2%)	11 (91.7%)	1.000	0.868
Yes	32 (12.9%)	7 (17.5%)	1.438 (0.587~3.524)		11 (9.8%)	1 (8.3%)	0.835 (0.098~7.092)	
**HBsAg**								
Negative	131 (52.6%)	20 (50.0%)	1.000	0.759	70 (62.5%)	7 (58.3%)	1.000	0.777
Positive	118 (47.4%)	20 (50.0%)	1.110 (0.569~2.165)		42 (37.5%)	5 (41.7%)	1.190 (0.355~3.991)	
**Anti-HCV**								
Negative	167 (67.1%)	28 (70.0%)	1.000	0.713	57 (50.9%)	10 (83.3%)	**1.000**	**0.032**
Positive	82 (32.9%)	12 (30.0%)	0.873 (0.422~1.804)		55 (49.1%)	2 (16.7%)	**0.207 (0.043~0.989)**	
**Liver cirrhosis**								
Negative	35 (14.1%)	8 (20.0%)	1.000	0.327	13 (11.6%)	1 (8.3%)	1.000	0.733
Positive	214 (85.9%)	32 (80.0%)	0.654 (0.279~1.536)		99 (88.4%)	11 (91.7%)	1.444 (0.172~12.121)	

ORs with their 95% CIs were estimated by logistic regression models. * *p* < 0.05 as statistically significant.
